# Supplementary material for: Causal Relationship between Aging and Anorexia Nervosa: A White-Matter-Microstructure-Mediated Mendelian Randomization Analysis
Source: Biomedicines. 2024 Aug 16;12(8):1874. doi: 10.3390/biomedicines12081874 (PMC11351342; doi:10.3390/biomedicines12081874)
Supplement: Supplementary file 1 [file biomedicines-12-01874-s001.zip › Supplementary Figure/Figure S4/Singe SNP plot of MR analysis for TL to ubm-b-1761.pdf]

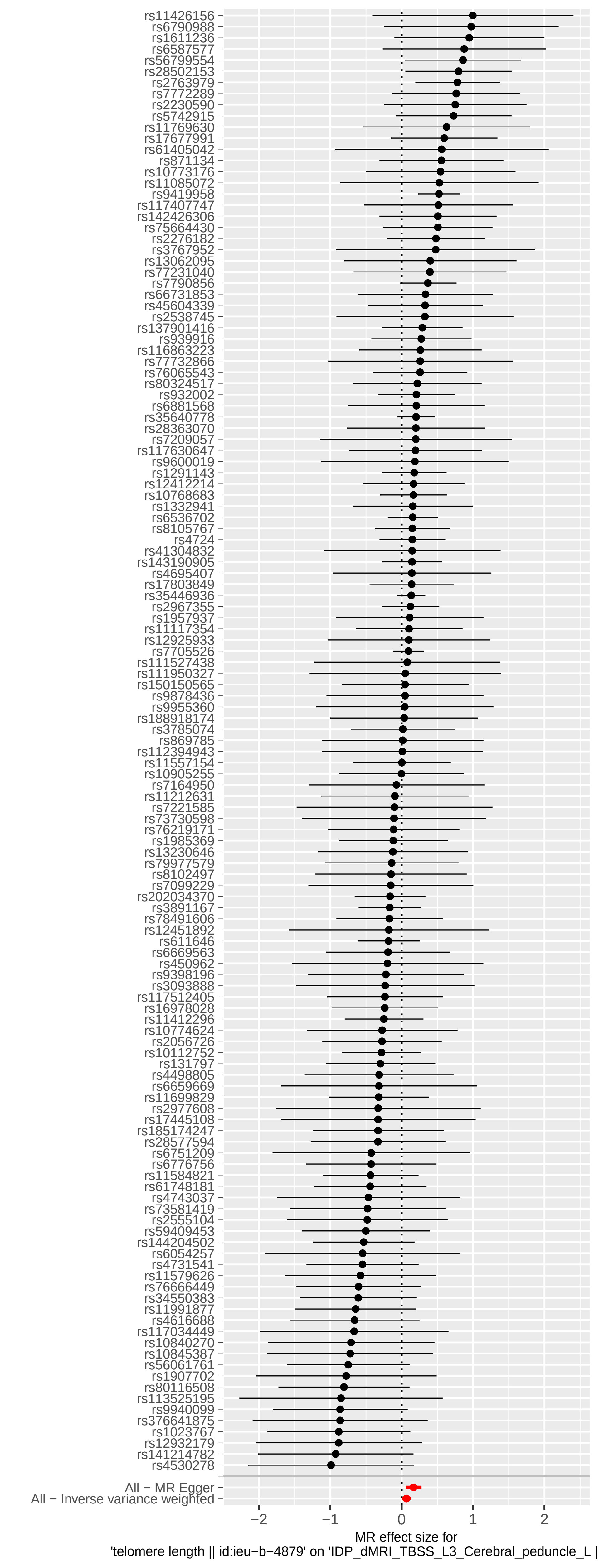
rs11426156
rs6790988
rs1611236
rs6587577
rs56799554
rs28502153
rs2763979
rs7772289
rs2230590
rs5742915
rs11769630
rs17677991
rs61405042
rs871134
rs10773176
rs11085072
rs9419958
rs117407747
rs142426306
rs75664430
rs2276182
rs3767952
rs13062095
rs77231040
rs7790856
rs66731853
rs45604339
rs2538745
rs137901416
rs939916
rs116863223
rs77732866
rs76065543
rs80324517
rs932002
rs6881568
rs35640778
rs28363070
rs7209057
rs117630647
rs9600019
rs1291143
rs12412214
rs10768683
rs1332941
rs6536702
rs8105767
rs4724
rs41304832
rs143190905
rs4695407
rs17803849
rs35446936
rs2967355
rs1957937
rs11117354
rs12925933
rs7705526
rs111527438
rs111950327
rs150150565
rs9878436
rs9955360
rs188918174
rs3785074
rs869785
rs112394943
rs11557154
rs10905255
rs7164950
rs11212631
rs7221585
rs73730598
rs76219171
rs1985369
rs13230646
rs79977579
rs8102497
rs7099229
rs202034370
rs3891167
rs78491606
rs12451892
rs611646
rs6669563
rs450962
rs9398196
rs3093888
rs117512405
rs16978028
rs11412296
rs10774624
rs2056726
rs10112752
rs131797
rs4498805
rs6659669
rs11699829
rs2977608
rs17445108
rs185174247
rs28577594
rs6751209
rs6776756
rs11584821
rs61748181
rs4743037
rs73581419
rs2555104
rs59409453
rs144204502
rs6054257
rs4731541
rs11579626
rs76666449
rs34550383
rs11991877
rs4616688
rs117034449
rs10840270
rs10845387
rs56061761
rs1907702
rs80116508
rs113525195
rs9940099
rs376641875
rs1023767
rs12932179
rs141214782
rs4530278
All − MR Egger
All − Inverse variance weighted
−2
−1
0
1
2
MR effect size for
'telomere length || id:ieu−b−4879' on 'IDP_dMRI_TBSS_L3_Cerebral_peduncle_L |
